# Program standards and student competencies among global chiropractic accreditation agencies: a content analysis

**DOI:** 10.1186/s12909-025-08052-3

**Published:** 2025-10-21

**Authors:** Claire D. Johnson, Bart N. Green, Lyndon Amorin-Woods, David Byfield, Waleska Crespo-Rivera, Philip Dewhurst, Chantale Doucet, Andy Dunn, Marina Fox, Amanda Jones-Harris, Carolina Kolberg, Charmaine M Korporaal, Craig Little, Daniel Moore, John Mrozek, Gary Schultz, Gregory Snow, Stephney Whillier, David Wickes, Yi Kai Wong, Christopher Yelverton, Igor Himelfarb

**Affiliations:** 1 National University of Health Sciences, Lombard, USA; 2 Scripps Health, San Diego, USA; 3 Murdoch University, Perth, Australia; 4 University of South Wales, Pontypridd, UK; 5 Universidad Central del Caribe, Bayamón, Puerto Rico; 6 Health Sciences University, Bournemouth, UK; 7 Université du Québec à Trois-Rivières, Trois- Rivières, Canada; 8 Veterans Affairs Western New York, Buffalo, USA; 9 New Zealand College of Chiropractic, Auckland, New Zealand; 10 Universidade Feevale, Novo Hamburgo, Brazil; 11 Durban University of Technology, Durban, South Africa; 12 Council on Chiropractic Education, Scottsdale, USA; 13 Teesside University, Middlesbrough, UK; 14 Texas Chiropractic College, Pasadena, USA; 15 University of Western States, Portland, USA; 16 Palmer College of Chiropractic West, San Jose, USA; 17 Life Chiropractic College West, Hayward, USA; 18 Macquarie University, Sydney, Australia; 19 Canadian Memorial Chiropractic College, Toronto, Canada; 20 School of Alternative and Complementary Medicine, IMU University, Kuala Lumpur, Malaysia; 21 University of Johannesburg, Johannesburg, South Africa; 22 National Board of Chiropractic Examiners, Greeley, USA

**Keywords:** Accreditation, Credentialing, Health occupations, Chiropractic, Education

## Abstract

**Background:**

Accreditation of healthcare provider training programs ensures graduate competency and provides a means for programs to improve. Accreditation consistency assures the public that healthcare providers have similar basic training across world regions. Currently, it is unknown if chiropractic accrediting agencies have congruent standards globally. Therefore, the purpose of this study was to investigate similarities and differences in student competencies and program standards among four chiropractic accreditation agencies worldwide.

**Methods:**

A quantitative content analysis was performed on accreditation standards from regional international accreditation agencies responsible for accrediting the majority of the world’s chiropractic degree programs. Agencies included the Council on Chiropractic Education (United States), the European Council on Chiropractic Education (Europe, United Kingdom, South Africa), the Council on Chiropractic Education Australasia (Australia, New Zealand, Malaysia), and the Council on Chiropractic Education Canada (Canada). The contents of the accrediting standards were coded using a standardized coding list. A modified Delphi technique was used by 21 international experts from December 1, 2023, to April 18, 2024. After four rounds of consideration to achieve consensus, the contents were analyzed for frequency and congruence of coded items across the accrediting agencies’ standards. A two-way analysis of variance was conducted to identify if there were any differences among the accreditation agencies.

**Results:**

Neither student competencies [*F*(3,8) = 0.007, *p* > .05] nor program standards [*F*(3,4) = 0.002, *p* > .05] differed significantly across the accrediting agencies. The statistical relationships between accreditation agencies and coding frequencies remained stable across all coded items, with no single code exhibiting differential performance depending on the accrediting body. The overall model showed *R*^*2*^ = 0.96 for student competencies and *R*^*2*^ = 0.87 for program standards; thus, the models’ predictions align with the observed data.

**Conclusions:**

The study findings demonstrate congruence for student competencies and program standards among chiropractic accreditation agencies across multiple geographic regions. The patterns of content were stable and consistent across the four accrediting agencies, with no evidence of differential effects among the agencies. In addition, this study provides essential details and standardized codes for agencies’ documents, which may facilitate dialogue and comprehension among agencies, educators, regulators, governing officials, and other stakeholders in chiropractic education.

**Study registration:**

The study protocol was prospectively registered with Open Science Framework on November 30, 2023 10.17605/OSF.IO/259WC.

**Supplementary Information:**

The online version contains supplementary material available at 10.1186/s12909-025-08052-3.

## Background

Accreditation agencies play a critical role in reassuring the public and jurisdictional licensing authorities that healthcare programs meet accepted standards for graduating practitioners who provide safe, effective, and competent care [1, 2]. Professional competence is required to function as a healthcare professional and is comprised of relevant knowledge, skills, behaviors, and values that can be measured against a set of standards [3, 4]. It has been noted that there is “a great variation in the education and training of physicians and nurses from country to country” [5] as well as variance in accreditation agencies throughout the world [6, 7]. The increasing internationalization of healthcare professions raises the issue that there is a need for quality assurance and a clear understanding of accreditation standards of education programs for all healthcare professionals [8].

Chiropractic is a healthcare profession with more than 100,000 practitioners [9, 10], who practice in 90 countries [11], and whose services are incorporated in both public and national healthcare systems [12–14]. Chiropractic is expanding, and the number of chiropractic education programs worldwide is increasing [8]. This growth in training programs is predicted to nearly double over the next 30 years [15, 16]. Yet despite this growth and subsequent contribution to the global healthcare workforce, chiropractic education programs, educational standards, and accreditation agencies remain understudied. Given the global growth of chiropractic, it would be beneficial for stakeholders to understand the variations in chiropractic education program requirements worldwide.

To identify existing published or in-process studies that systematically inventoried international chiropractic accrediting body documents, a literature search was performed in November 2023. We searched MEDLINE (PubMed), the Cumulated Index to Nursing and Allied Health Literature (CINAHL via EBSCO), Index to Chiropractic Literature (ICL), and Educational Resources Information Center (ERIC). We also searched the International Prospective Register of Systematic Reviews (PROSPERO) and Open Science Framework. Studies comparing variations in accreditation for other health professions were found [17–20], whereas no similar studies exist for chiropractic. Although prior studies described several of the chiropractic accreditation agencies [21–23], no systematic studies of current chiropractic accrediting agency standards have been published. This knowledge gap negatively affects communication and comprehension among stakeholders, including accrediting agencies, regulatory bodies, policymakers, qualifying boards, program administrators, faculty, students, practitioners, patients, and the public.

Therefore, the primary purpose of this study was to perform a content analysis to investigate similarities and differences in program standards and student competencies among four chiropractic accreditation agencies worldwide. The secondary purpose was to demonstrate the application of a coding document that describes and quantifies concepts related to program standards and student competencies, with the aim of facilitating dialogue among stakeholders about global chiropractic education standards.

## Methods

A modified Delphi consensus process was performed to code the contents of accreditation documents. Following consensus, a two-way analysis of variance (ANOVA) was performed to identify whether coding differed significantly among the four accrediting agencies. This study was deemed exempt by the National University of Health Sciences Institutional Review Board (RS2301). The need for consent to participate was waived by the National University of Health Sciences Institutional Review Board. This study adhered to the Declaration of Helsinki.

### Characteristics of the panelists

A geographically diverse panel of experts was invited to reach a consensus opinion on the coding of the accreditation document contents prior to analysis. Panelists with expertise in chiropractic education were selected to represent four world regions and eight countries where chiropractic programs are accredited, thereby providing a balanced perspective to inform the decision-making process for this study. Countries represented were Australia, Brazil, Canada, Malaysia, New Zealand, South Africa, the United Kingdom, and the United States. Combined, the panelists represent the countries that comprise an estimated 92% of the world’s chiropractors [9]. Panelists and authors were not remunerated for participating in the study.

### Coding process

In June 2023, the current versions of publicly available standards and competencies documents from four chiropractic accreditation agencies were retrieved from publicly available sources on the Internet. The four agencies selected were the Council on Chiropractic Education (CCE), the European Council on Chiropractic Education (ECCE), the Council on Chiropractic Education Australasia (CCEA), and the Council on Chiropractic Education Canada (CCEC). The CCE is recognized by the United States Department of Education and the Council for Higher Education Accreditation as the accrediting body for chiropractic programs [24]. The 2021 version of the CCE standards was used for this study. The ECCE is an international, autonomous organization established by the chiropractic profession in Europe to accredit programs that provide first-qualification chiropractic education and training [25]. The 2019 version of the ECCE accreditation standards was used for this study. The CCEA is appointed by the Chiropractic Board of Australia and the New Zealand Chiropractic Board as the independent accrediting authority for chiropractic education [26]. The 2017 version of the CCEA accreditation standards was used for this study. The CCEC is a committee of the Federation of Canadian Chiropractic and makes the accreditation decisions for that body [27]. The 2018 version of the CCEC accreditation standards was used for this study. The four agencies accredit 38 institutions, which represent the majority of chiropractic programs worldwide (see Supplemental File Appendix A).

We selected ECCE, which is a member of the Councils on Chiropractic Education International (CCEI) for the European region. Whereas the General Chiropractic Council (GCC) regulates chiropractors only in the United Kingdom, the ECCE includes accreditation in the UK, as well as in Europe and South Africa, thereby covering a broader range of global regions. At the initiation of this project, the Council on Chiropractic Education—Latin America (CCE-LA) was in development but had not yet accredited any chiropractic programs in Latin America; therefore, it was not included.

Content was extracted from the accreditation documents (by CDJ) and listed in two spreadsheets: one for student competencies (with 249 items in total) and one for program standards (with 136 items in total). We coded text from the documents by systematically analyzing text data by assigning predefined codes to segments of text. A predefined set of codes was used to classify the text data (see supplemental file Appendix B) [28, 29]. The definitions and examples for each code were provided during the coding process to ensure consistency in coding.

For the first three modified Delphi rounds, panelists were instructed to review each accreditation statement and indicate whether they agreed with the provided codes. Panelists read through the text data, line by line, and noted the appropriate code for each item based on the predefined coding scheme. Coding was done manually; we did not use qualitative data analysis software. To ensure consistency in coding, multiple coders independently coded the same data, and the results were compared. The purpose of coding was to categorize the content represented in each of the accreditation documents and to verify the accuracy of the coding through a modified Delphi consensus process. All panelists had participated in the development and training for the coding tool as part of a previous study, so the coding procedure was not piloted again [29].

Panelists were instructed that the code should only be related to the content of the text, not to extrapolate meaning, which should be obvious without any additional explanation. They were informed that although they may not agree with how some items were written, the purpose of this study was not to critique, correct, or rewrite the content. Their task was only to code what was stated in each item of the accreditation text. Because many statements were complex, more than one code was allowed per item.

If the panelist did not agree with the code provided, they were asked to type in a suggested correction and provide a rationale for the proposed change, which would be included in the next round of review. Panelists were informed that their responses would be collated anonymously, the codes would be amended according to group input and then returned to the group if another review round was necessary. Panelists were given additional space on the form to provide comments about the overall process or share any additional insights about the text that they were coding.

Two to three rounds of review were prospectively planned, which is a commonly reported number for Delphi studies [30]. We defined agreement as the panelists agreeing to the codes of an individual item without any comments or corrections. A consensus agreement was decided a priori as 80%. This is higher than the commonly accepted threshold of 75% and falls within the range of 50–97% reported in other studies [31–33]. For items for which all panelists had no corrections or suggestions, we considered this to be 100% agreement. Appendix C in the supplemental file describes each step and the consensus reached through the agreement process.

All 21 panelists participated in each of the three modified Delphi rounds and the consensus panel. Demographic information about the participants can be found in Appendix D of the supplemental file. Blinded communications were sent to the panelist group to inform them of each step of the process. Round one was completed from December 1 to December 11, 2023. Rounds two and three were completed by January 15, 2024, and by April 14, 2024, respectively. The Round 4 nominal group technique was completed on April 18, 2024. All panelists participated, and there were no deviations from the study protocol. The percent agreements for coding for each round are represented in the Appendix E supplemental file.

### Statistical analysis

To examine whether student competencies and program standard content were significantly different across accreditation agencies, a two-way analysis of variance (ANOVA) was conducted using IBM SPSS Statistics (version 31) [34–36]. An analysis was performed with variables Accreditation Agency (CCE, CCEA, ECCE, and CCEC) and Student Competencies (A1 through D2). In a second analysis, the variables were: Accreditation Agency (CCE, CCEA, ECCE, and CCEC), and Program Code (E1 through E10).

The two-way ANOVA was performed on additive data; therefore, the analysis allowed for the evaluation of the main effects but not the interaction. The main effect of the accreditation agency was whether coding differed significantly among the four accrediting agencies regardless of the code. The main effect of the code assessed whether certain agencies consistently had a greater or smaller proportion of coded items, regardless of the accrediting agency.

Before conducting the two-way ANOVA, standard assumptions were tested to ensure the validity and robustness of the model. Normality of residuals was assessed using the Shapiro–Wilk test, and distributional patterns were further examined through Q–Q plots. Homogeneity of variances across groups was evaluated using Levene’s test. The independence of observations was presumed based on the structure of the study’s design and the data collection methodology. Following confirmation of assumptions, the two-way ANOVA was performed. Where significant main effects were observed, Least Significant Difference (LSD) post-hoc tests were conducted to explore specific pairwise differences among group means. For statistical formulas, see Appendix F in the supplemental file.

## Results

### Coding results

There were 1086 student competency codes overall, represented by CCE (353), CCEA (314), ECCE (93), and CCEC (326), with an average of 4.36 codes per student competency. The program standards had 248 codes, represented by CCE (80), CCEA (64), ECCE (69), and CCEC (35), with an average of 1.82 codes per program standard. For the full description of each code, please see Appendix B in the supplemental file.

### Student competencies

The codes were normally distributed across groups, as confirmed by the Shapiro–Wilk test and Q–Q plots. (See Appendix F in the supplementary file) Levene’s test supported the homogeneity of variances across groups. No significant main effect was found among accreditation agencies, *F*(3,8) = 0.007, *p* >.05, indicating that the accrediting body, whether CCE, CCEA, ECCE, or CCEC, had no measurable difference regarding student competencies. All four agencies demonstrated comparable outcomes across student competencies. The $\:{R}^{2}$ associated with the model was 0.96, indicating that the model accounted for 96% of the variability in the data.

**Fig. 1:**
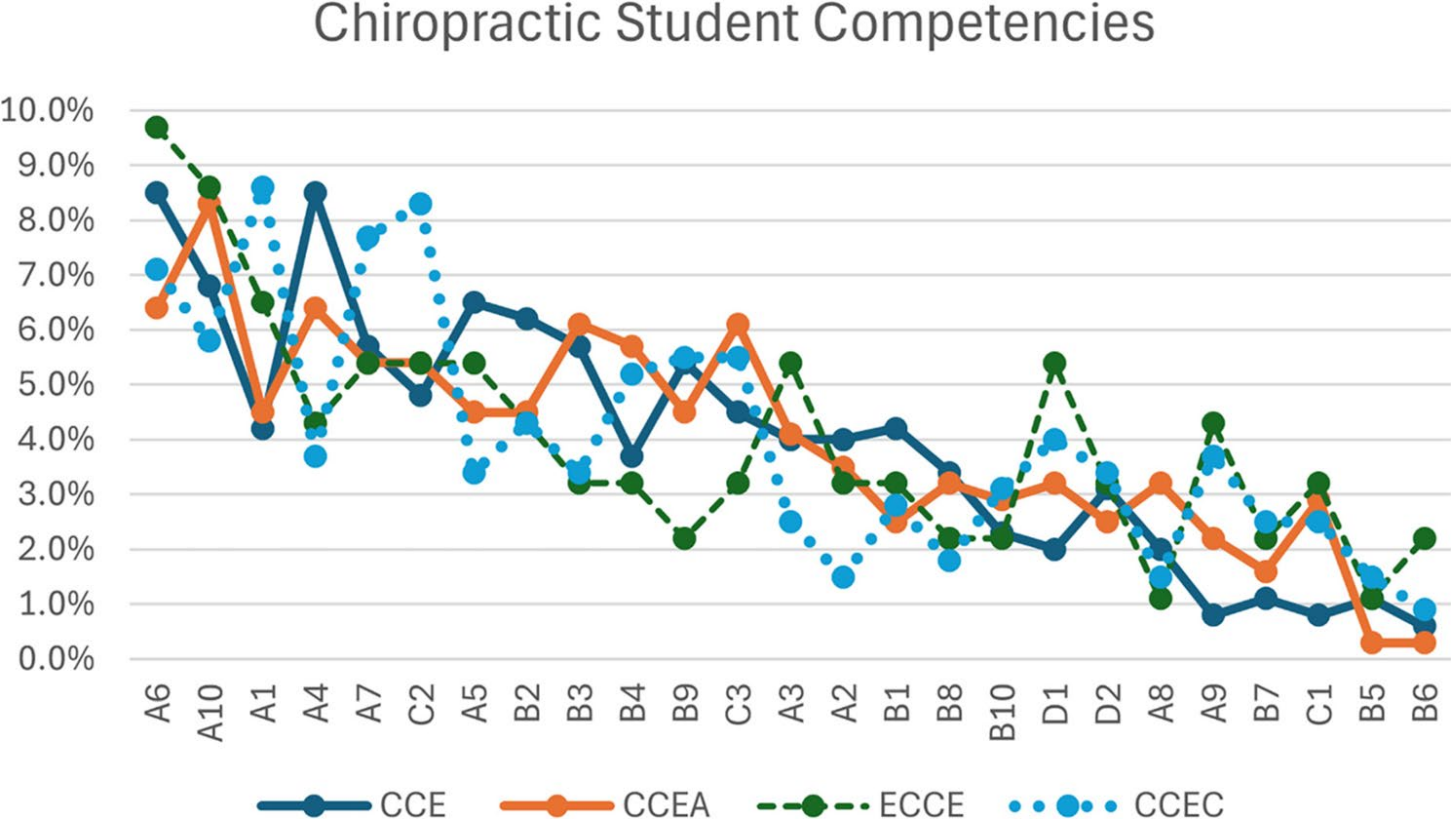
Proportion of student competency items for individual accrediting agencies from most common to least common (left to right)

To further investigate where specific agency differences may lie, Least Significant Difference (LSD) post-hoc tests were conducted. These analyses revealed multiple statistically significant pairwise differences among coded items (Fig. 1; Table 1). The codes were represented similarly (clustered) among the accreditation agencies. Code A1 (knowledge of chiropractic) reported significantly higher frequency than A2, A3, A8, A9, B1, B4, B5, and several others (*p* values ranging from < 0.001 to 0.048). Similarly, A6 (knowledge of chiropractic care) was significantly more frequent than others such as A2, A3, A5, and B1, among others (*p* <.05).

**Table 1:** Model results for student competencies

Source	Sum of Squares	df	F	*p*-value	Partial η²	ω²
Code	0.032	22	9.02	< .001	0.73	0.59
Agency	0.004	3	0.001	.99997	0.001	0.01
Residual	0.012	74				

### Program standards

**Fig. 2:**
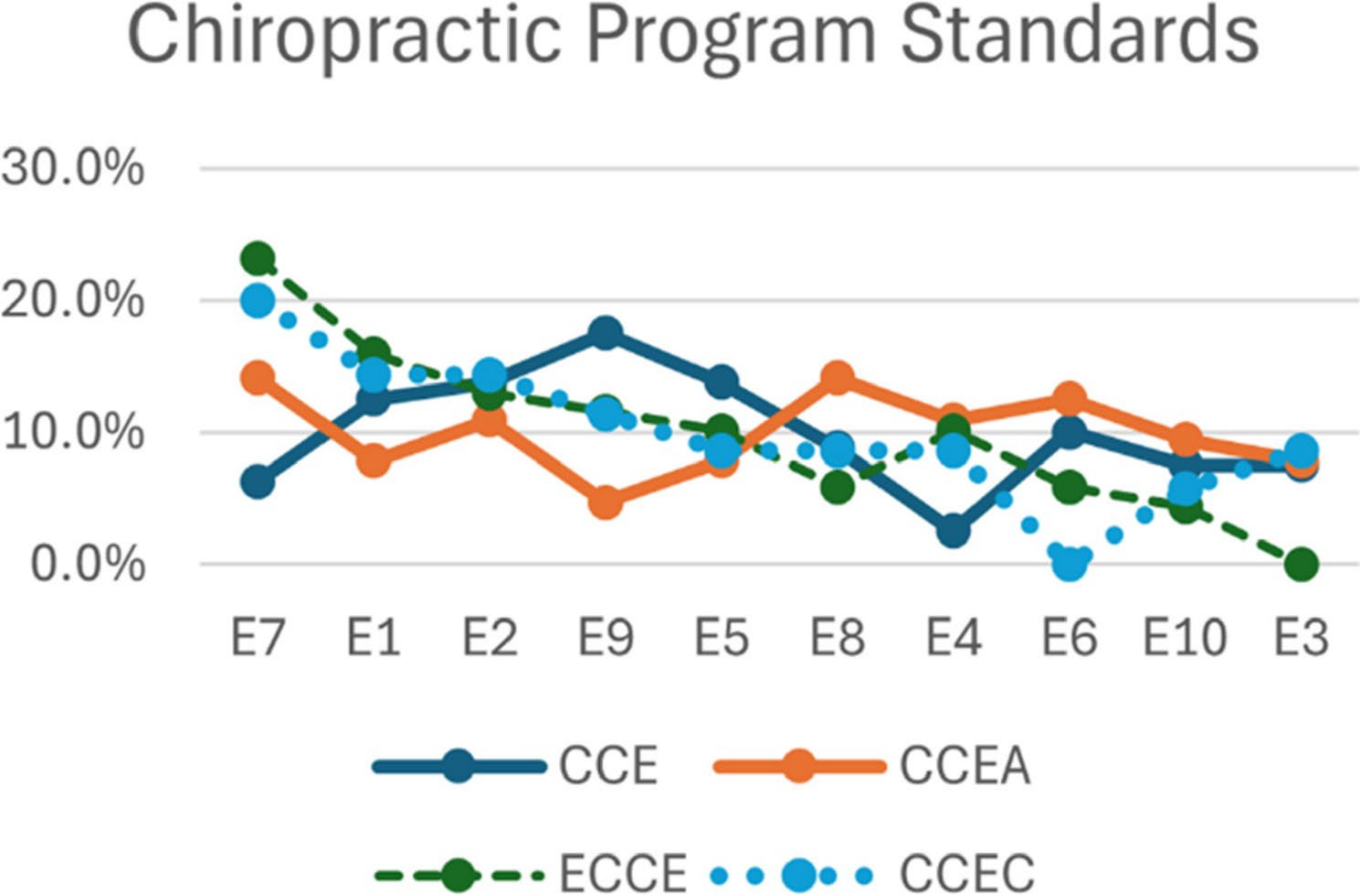
Proportion of program items for individual accrediting agencies from most common to least common (left to right)

No statistically significant main effects were identified for accreditation agency and program standard codes (E1–E10). Specifically, the effect of accreditation agency on program standards was not significant, *F*(3,4) = 0.002, *p* >.05, indicating that program standards did not differ across the four accrediting agencies. Similarly, the main effect of code was not significant, *F*(8,4) = 1.268, *p* >.05, suggesting that no individual program standard demonstrated codes that differed significantly from others (Fig. 2; Table 2). The overall model explained 87% of the variability in the data, $\:{R}^{2}$= 0.87. For codes, see Appendix G in the supplemental file.

**Table 2:** Model results for program standards

Source	Sum of Squares	df	F	*p*-value	Partial η²	ω²
Code	0.03	8	1.71	.141	0.33	0.21
Agency	0.001	3	0.0001	.99999	0.0001	0.001
Residual	0.012	28				

## Discussion

Accreditation is an essential process that provides the public assurance that healthcare programs provide high-quality education and graduate practitioners who can provide competent, safe, and effective care [2]. Prior studies have described several of the chiropractic accreditation agencies [21–23]. However, none of these have provided a detailed, systematic analysis of all content across global agencies. This study is the first of its kind to conduct a consensus process for categorizing chiropractic program standards and student competencies among four primary chiropractic accreditation agencies worldwide.

The analysis of accreditation agency documents revealed no statistically significant differences in chiropractic student competencies and program standards, confirming consistency among these four accreditation agencies. Although the documents from each of the accreditation agencies were formatted differently, they all contained proportionally similar content across coded items for this study. This congruence is important for stakeholders who consider expectations for chiropractic education and training across global regions. The proportional coding for each agency represents the global impression of the competencies and program standards that are included and emphasized worldwide. This may help those who are unfamiliar with chiropractic education better understand the focus and content of chiropractic programs. Additionally, this coding model can serve as a helpful framework for program administrators when developing a new chiropractic program or enhancing the quality of an existing one.

One may wonder why one accreditation agency has a disproportionate percentage of coded content compared to others. Prudence should be exercised when interpreting the proportion of coded items. A code’s frequency does not imply its value or importance. A code used only once may be very important and thus does not need to be mentioned more than once. Thus, simply because coded items are presented fewer times than others does not mean that they are less valuable than codes used multiple times. However, there may be reasons why content is present more or less frequently, which is further discussed here.

If a topic is less frequently included compared to others, the topic may still be emerging and not yet fully integrated by the accreditation agency. New concepts or paradigms may take decades to fully integrate into higher education and thus may appear less frequently. Another hypothesis is that the topic is already well-represented, so it may not require additional attention. For example, the competency for chiropractic manipulation (e.g., B5) is already a core component of chiropractic curricula; therefore, including this competency multiple times would seem redundant.

If a topic is found more frequently in one region than another, it is possible that there are regional variations and emphasis on requirements, which could explain the frequency differences between agencies. It is possible that regional laws and regulations governing higher education and the chiropractic profession may require the inclusion of a specific topic. Therefore, content may need to be emphasized in multiple places to ensure compliance. There may be regional, sociological, cultural, or historical events that create a heightened awareness of a topic, thereby creating a perceived need to revisit a concept. Additionally, duplication of content may have occurred to clarify a concept, or it is possible that the authors were simply unaware of the duplicated content.

Some may question whether accreditation standards should be identical for all regions. However, due to regional requirements in regulation and legislation, some variance among the accreditation documents is not only expected but practical. Chiropractic program accreditation standards have evolved over time, some beginning decades before others, and standards are constructed to be relevant to the jurisdictions that they serve. It is essential to acknowledge that regional differences exist in terms of needs, regulations, scope of practice, and terminology. Therefore, it should not be expected that all accreditation documents would be identical to each other.

### Future studies and recommendations

This content analysis may serve as a working document to clarify concepts within chiropractic accreditation and facilitate dialogue among stakeholders about similarities and differences of global chiropractic education standards. Follow-up studies could examine how accreditation agencies enforce their standards in comparison to one another, which may influence the academic outcomes of the institutions they accredit. The coding tool has potential use in future studies, both in chiropractic education and other disciplines. The coding tool document may serve as a starting point for new accreditation agencies in other world regions when developing standards, as well as a reference for existing agencies when revising their standards. Finally, this research provides a baseline indicator of the student competencies and program requirements necessary to produce competent chiropractic graduates worldwide. The methods used here may be used in subsequent content analyses to track trends over time.

We recommend the following when developing and revising accreditation standards. To be effective, accreditation agency documents must be clearly understood by educators and other stakeholders. However, during this study, we identified components in the accreditation documents that were unclear. Some content was repetitive, overly complex, or had confusing prose, resulting in comprehension difficulties. Therefore, we offer the following recommendations to assist accreditation agencies in revising their documents in the future.

1. Edit statements for clarity. Avoid statements with context-sensitive language. Avoid being too detailed, complex, or too general. Incorporate clarifying statements as needed to ensure that meaning and messaging are comprehensible.
2. Use common language and clarify terminology to improve understanding across regions. Avoid the use of general or nonspecific terms, which may cause difficulty with interpretation.
3. Focus on coherence, structure, and simplicity to enhance clarity and understandability. Avoid verbosity and repetitive language. Avoid compound statements that may be too complex to convey meaning clearly.

### Strengths

One strength of this study was the substantial representativeness of the authors. Every regional accreditation agency and all world regions with chiropractic education programs had representation on the panel. With a combined academic experience of 483 years (averaging 23 years per author), the panel’s subject matter expertise was rich and robust. Another strength was that we used a standardized coding tool; all accreditation documents were categorized using the same criteria. The strength and percent agreement per code were maintained, with 100% of the 21 panelists completing the entire process. Anonymization was maintained during the modified Delphi rounds, reducing the potential for bias by the influence of specific panel members. Another strength was that the student competencies and program standards were presented within the context of the entire accrediting agency document, thereby reducing the potential for standards to be interpreted out of context.

Our approach to content analysis, which utilizes a coding tool, consensus process, and statistical analysis, appears to be unique. The combination of methods reduces bias and provides confidence in the interpretation of the results. We hope that educators from other health professions consider using these methods.

### Limitations

Accreditation documents are living documents that are continually reviewed and periodically revised. Thus, our results represent the versions of accreditation standards available at the time of the study. However, unless an agency profoundly revises its standards, it is not expected that the main patterns noted here will be drastically different. Each world region has different requirements and regulations for health professions and higher education; thus, some discrepancies may be attributed to these regional variations. Two accrediting agencies (GCC and CCE-LA) were excluded from this study for reasons mentioned previously. No patients or other healthcare providers were included on the panel. Thus, if a different group of panelists had participated, there may have been additional or different findings. As with any lengthy document review, the number of items to be coded could have caused rating fatigue. However, panelists were provided with ample time to complete their work, and they could review and re-review their responses before submitting their completed sheets. We believe the use of 21 panelists reduced the possibility of coding errors, such that if one person missed an error, another would have identified it.

We acknowledge a limitation that the model did not include within-cell replication; thus, the interaction effect cannot be formally tested under the classical ANOVA framework. However, we retained the simplified additive model for descriptive purposes; thus, inferential claims regarding interactions are not possible.

Each region has its own vernacular; therefore, some terms may have been misunderstood by panelists from different regions. However, we included education experts from each region to help mitigate comprehension issues. Reviewers coded the content but did not delve into the creators’ intentions when crafting their statements. Thus, the results were pragmatic and reflected the panelists’ understanding of each coded item. We acknowledge that the coding tool may have its own limitations. The coding tool captures high-level content and not detailed information. Therefore, details about specific items were not provided in the coding tool. The purpose of coding was to identify the general topic category, allowing for further evaluation or detailed exploration.

## Conclusion

The findings of this content analysis demonstrate consistency among chiropractic accreditation agencies across multiple geographic regions in terms of student competencies and program standards. The patterns of coded content were stable and consistent across the four accrediting agencies, with no evidence of differential effects among the agencies. This congruence is essential when considering requirements for chiropractic education across global regions. This document also includes essential details and codes for the contents of agencies’ documents, which will help to clarify concepts within chiropractic education and facilitate dialogue and comprehension among stakeholders of chiropractic education.

## Supplementary Information

Supplementary Material 1.

## Data Availability

The data that support the findings of this study are available on reasonable request from the corresponding author.

## Abbreviations

CCE: Council on Chiropractic Education
ECCE: European Council on Chiropractic Education
CCEA: Council on Chiropractic Education Australasia
CCEC: Council on Chiropractic Education Canada
GCC: General Chiropractic Council
CCE-LA: Council on Chiropractic Education—Latin America
ANOVA: Analysis of variance
