# Supplementary material for: Program standards and student competencies among global chiropractic accreditation agencies: a content analysis
Source: BMC Med Educ. 2025 Oct 21;25:1473. doi: 10.1186/s12909-025-08052-3 (PMC12542019; doi:10.1186/s12909-025-08052-3)
Supplement: Supplementary file 1 — Supplementary Material 1. [file 12909_2025_8052_MOESM1_ESM.pdf]

Supplementary file

Program Standards and Student Competencies among Global Chiropractic Accreditation Agencies: A Content Analysis

Appendix A - chiropractic programs, regions, and the accreditation agencies
Appendix B - coding tool, descriptions, and examples
Appendix C - description of the modified Delphi consensus process
Appendix D – demographics of panelists/authors
Appendix E - percent agreements
Appendix F - statistical formulations
Appendix G – code frequencies

AUTHORS
Claire D Johnson; National University of Health Sciences, Lombard, United States; and Scripps Health, San Diego, United States
Bart N Green; National University of Health Sciences, Lombard, United States; and Scripps Health, San Diego, United States
Lyndon Amorin-Woods; Murdoch University, Perth, Australia
David Byfield; University of South Wales, Pontypridd, United Kingdom
Waleska Crespo-Rivera; Universidad Central del Caribe, Bayamón, Puerto Rico
Philip Dewhurst; Health Sciences University, Bournemouth, United Kingdom
Chantale Doucet; Université du Québec à Trois-Rivières, Trois- Rivières, Canada,
Andy Dunn; Veterans Affairs Western New York, Buffalo, United States
Marina Fox; New Zealand College of Chiropractic, Auckland, New Zealand
Amanda Jones-Harris; AECC University College, Bournemouth, United Kingdom
Carolina Kolberg; Universidade Feevale, Novo Hamburgo, Brazil
Charmaine M Korporaal; Durban University of Technology, Durban, South Africa
Craig Little; Council on Chiropractic Education, Scottsdale, United States
Daniel Moore; Teesside University, Middlesbrough, United Kingdom
John Mrozek; Texas Chiropractic College, Pasadena, United States
Gary Schultz; University of Western States, Portland, United States
Gregory Snow; Palmer College of Chiropractic West, San Jose, United States; and Life Chiropractic College West, Hayward, United States
Stephney Whillier; Macquarie University, Sydney, Australia,
David Wickes; Canadian Memorial Chiropractic College, Toronto, Canada
Yi Kai Wong; IMU University, Kuala Lumpur, Malaysia
Christopher Yelverton; University of Johannesburg, Johannesburg, South Africa
Igor Himelfarb; National Board of Chiropractic Examiners, Greeley, United States

Appendix A

The following table includes chiropractic programs, regions, and the accreditation agencies that are represented in this study.

Accrediting Agency	Accredited Chiropractic Program	Country	Year program was founded	Website	World Region
CCE	Campbellsville University	United States	2021	www.campbellsville.edu/	RoA
CCE	Cleveland University	United States	1922	www.cleveland.edu	RoA
CCE	D'Youville College	United States	2004	www.dyc.edu	RoA
CCE	Keiser University	United States	2016	www.keiseruniversity.edu	RoA
CCE	Life Chiropractic College West	United States	1981	www.lifewest.edu	RoA
CCE	Life University	United States	1974	www.life.edu	RoA
CCE	Logan University	United States	1935	www.logan.edu	RoA
CCE	National University of Health Sciences (Illinois & Florida)	United States	1906	www.nuhs.edu	RoA
CCE	Northeast College of Health Sciences	United States	1919	www.northeastcollege.edu	RoA
CCE	Northwestern Health Sciences University	United States	1941	www.nwhealth.edu	RoA
CCE	Palmer College of Chiropractic Davenport	United States	1897	www.palmer.edu	RoA
CCE	Palmer College of Chiropractic Florida	United States	2002	www.palmer.edu	RoA
CCE	Parker University	United States	1978	www.parker.edu	RoA
CCE	Sherman College of Chiropractic	United States	1973	www.sherman.edu	RoA
CCE	Southern California University of Health Sciences	United States	1911	www.scuhs.edu	RoA
CCE	Texas Chiropractic College	United States	1908	www.txchiro.edu	RoA
CCE	University of Bridgeport College of Chiropractic	United States	1991	www.bridgeport.edu/chiro	RoA
CCE	University of Western States	United States	1904	www.uws.edu	RoA
CCE	Universidad Central del Caribe	Puerto Rico	2018	www.uccaribe.edu	RoA
CCEA	Australian Chiropractic College	Australia	2016	https://acc.sa.edu.au	WPR
CCEA	Central Queensland University (CQU University, Brisbane)	Australia	2012	www.cqu.edu.au	WPR
CCEA	Macquarie University	Australia	1990	www.chiro.mq.edu.au/Clinics	WPR
CCEA	Murdoch University	Australia	2006	www.chiropractic.murdoch.edu .au	WPR
CCEA	International Medical University (IMU University)	Malaysia	2010	www.imu.edu.my	WPR
CCEA	New Zealand College of Chiropractic	New Zealand	1994	www.chiropractic.ac.nz	WPR
CCEC	Université du Québec à Trois-Rivières	Canada	1992	www.uqtr.ca	RoA
CCEC, CCE	Canadian Memorial Chiropractic College	Canada	1945	www.cmcc.ca	RoA
ECCE	Durban University of Technology	South Africa	1989	www.dut.ac.za	AR
ECCE	University of Johannesburg	South Africa	1993	www.uj.ac.za	AR
ECCE	Health Sciences University - AECC School of Chiropractic	United Kingdom	1965	www.aecc.ac.uk	ER
ECCE	London South Bank University	United Kingdom	2018	www.lsbu.ac.uk	ER
ECCE	McTimoney College of Chiropractic (Oxfordshire & Manchester)	United Kingdom	1972	www.mctimoney-college.ac.uk	ER

ECCE	University of South Wales - Welsh Institute of Chiropractic	United Kingdom	2001	www.southwales.ac.uk/	ER
ECCE	Syddansk Universitet Odense	Denmark	1994	www.sdu.dk	ER
ECCE	Institut Franco-Européen de Chiropratique (Paris & Toulouse)	France	1983	www.ifec.net	ER
ECCE	Barcelona College of Chiropractic	Spain	2009	www.bcchiropractic.es	ER
ECCE	Madrid College of Chiropractic-RCU	Spain	2007	www.rcumariacristina.com	ER
ECCE	University of Zurich	Switzerland	2008	www.balgrist.ch	ER

RoA = Region of the Americas
WPR = Western Pacific Region
AR = African Region
ER = European Region
CCE = Council on Chiropractic Education
CCEA = Council on Chiropractic Education Australasia
ECCE = European Council on Chiropractic Education
CCEC = Council on Chiropractic Education Canada
Source of data www.WFC.org, www.ccea.com.au, www.cce-europe.com, www.chirofed.ca, www.cceintl.org, www.cce-usa.org.

Chiropractic programs not represented because they were in regions without active chiropractic program accreditation agencies, or had other approval/recognition, or were a developing program (not yet accredited) are as follows: Bahçeşehir Üniversity - Chiropractic Program; Universidade Anhembi Morumbi; Universidade Feevale; Centro Universitario: Unidade Central de Educação Faem Faculdade; Centro Universitario: Gama e Souza Centro: Universitario Fametro, Universidad Central de Chile; Universidad Estatal del Valle de Ecatepec; Universidad Estatal del Valle de Toluca; Universidad Veracruzana; Teesside University; Skandinaviska Kiropraktorhögskolan; University of Pittsburgh doctor of chiropractic program. The following training programs were also excluded because of closure: RMIT University; Palmer College of Chiropractic West.

Appendix B

Coding tool, which includes the codes, their descriptions, and examples (Published with permission from Brighthall.)

	Student Competencies
Code	Student Knowledge, Cognitive Competence
	Knowledge/cognitive competence "the possession of appropriate work-related knowledge, the ability to put this to effective use."
A1	Knowledge of chiropractic (principles, theories, history, how chiropractic fits in to the healthcare system) Examples include understanding chiropractic as a profession, identifying professional behaviors, chiropractic principles and practices, chiropractic theories, history of chiropractic and healthcare, philosophy of chiropractic, professional traits that are unique to chiropractic, biopsychosocial model of health, and how chiropractic contributes to the health of patients and populations.
A2	Knowledge of normal (structure and function, anatomy, physiology, biokinematics, biology, chemistry, physiology, biochemistry, immunology, genetics and microbiology, biopsychosocial, foundational knowledge etc) Examples include comprehending anatomy, bio-kinematics, biology, biochemistry, physiology, immunology, genetics, histology, microbiology, health-related biopsychosocial factors, metrics for normal health, and foundational knowledge, history and exam findings of normal structure and function.
A3	Knowledge of abnormal (structure and function, pathology, dysfunction, disease process, microbiology, biopsychosocial, foundational knowledge, etc) Examples include knowing pathology, biomechanical dysfunction, disease processes, communicable and noncommunicable diseases, comorbidities, microbiology, harmful biopsychosocial factors, foundational knowledge, history and exam findings for abnormal structure and function.
A4	Knowledge of health (determinants of health, disease prevention, health promotion, public health, nutrition, physical activity, injury prevention, etc) Examples include understanding social determinants of health, injury prevention, disease prevention, health promotion, public health, diet/nutrition, physical activity, injury prevention, wellness/wellbeing, and community health.
A5	Knowledge of evaluation (information needed for patient assessment, neurology, orthopedics, clinical findings, patient management, diagnostic tests, diagnostic imaging, laboratory, clinical evaluation, etc) Examples include understanding information needed for patient assessment, including history taking, physical examination, neurologic and musculoskeletal evaluation, clinical findings, patient management, diagnostic tests, diagnostic imaging, laboratory, and clinical evaluation.
A6	Knowledge of chiropractic care (information related to safety and effects of chiropractic care, manipulation, modalities, management, supportive active and passive treatment methods, etc) Examples include comprehending common chiropractic standard treatment protocols, treatment modalities (adjustment/manipulation, manual and other modalities, education, rehabilitation, active care, and therapeutic care), emergency care, patient management, supportive active and passive treatment methods, safety, and effects of chiropractic care.
A7	Knowledge of patient/person needs (characteristics of patient choices/needs, values, diversity/equity, pain, healing process and relationships, etc) Examples include knowing specific individual characteristics of patients that impact care, patient needs/values/preferences/autonomy, patient self-efficacy, cultural differences, diversity/equity/inclusion, shared decision making, perceptions of pain, healing process, boundaries, therapeutic relationships, person-centered, and people-centered knowledge.
A8	Knowledge of special populations (emergency care, pediatrics, geriatrics, sports, men/women's health, workers, etc) Examples include knowing specific characteristics related to person-centered and people-centered care, care of youth, older adults, athletes, men's/women's health, workplace injuries and safety, health needs throughout the life course, and care of people with disabilities.
A9	Knowledge of research (fundamental concepts about critical appraisal and application of research and science related to health care) Examples include comprehending the foundations of chiropractic-relevant and healthcare research and science, fundamental concepts about searching for trustworthy scientific information, critical appraisal, and use of best evidence, and identifying and applying new knowledge to individual patients within the healthcare setting.
A10	Knowledge of other factors (clinical impacts and implications of concurrent health conditions, other health professions' treatments, or other factors that may impact chiropractic care) Examples include knowing how chiropractic functions within healthcare systems and health services, clinical impacts, and implications of concurrent management of patients with other providers or patients' self-initiated care, other health professions' common standard treatment protocols, other models of care such as the biomedical model, other factors that may impact patients receiving chiropractic care, patient access to care, integrated care delivery, and legal, regulatory or other requirements for a chiropractor to work effectively within the healthcare system.
Code	Student Functional Competence
	Functional competence "the ability to perform a range of work-based tasks effectively to produce required outcomes."
B1	Perform assessment of the individual patient (patient-relevant history, examination, order/refer tests such as lab, diagnostic tests, other providers) Examples include performing a patient-relevant history, examination, palpation/physical assessment procedures, health screening, ordering or referring a patient for appropriate tests such as lab, diagnostic tests, diagnostic imaging, or to other providers for additional assessment.
B2	Demonstrate clinical reasoning skills for the individual patient (formulate diagnosis/clinical impression, differential diagnosis, make a therapeutic decision, estimate prognosis for the patient) Examples include demonstrating clinical reasoning about the patient, formulating a diagnosis/clinical impression, differential diagnoses, making a therapeutic decision, estimating prognosis for the patient, and determining safety of treatments for patients.

**B3**	Demonstrate health management (development of management plan, monitor patient response to care, coordinated care, referral) Examples include developing a management plan for patient care, preventive care, monitoring patient response to care, coordinating care, referring to other providers when indicated, and releasing the patient from care when appropriate.
**B4**	Perform communication (report of findings, obtain patient consent, discuss patient values, expectations of treatment plan, prognosis) Examples include communicating throughout the clinical encounter, history taking, report of findings, informed consent process, discuss patient values, communicating expectations of a treatment plan, prognosis, shared decision-making, and instructions for caregivers.
**B5**	Perform manual therapies (adjusting /manipulation/ mobilization, manual soft tissue therapies) Examples include implementing manual or instrument assisted spinal and extremity adjusting /manipulation /mobilization, manual and instrument assisted soft tissue therapies.
**B6**	Perform supporting therapies (non-manipulative) (e.g., modalities, exercise/active therapies, rehabilitative therapies) Examples include performing physical modalities (e.g., ultrasound, electrotherapy), exercise/active therapies, rehabilitative therapies, and taping/bracing.
**B7**	Demonstrate therapeutic education, instructions (e.g., self-care, home exercises, diet/nutrition, injury prevention, etc) Examples include demonstrating therapeutic education, instructions for self-care, home exercises, diet/nutrition, instructions about rehabilitation, injury prevention, and ergonomics.
**B8**	Demonstrate safety (patient safety, provider safety, hygiene, risk reduction in the clinical setting) Examples include showing patient safety, provider and staff safety, hygiene, risk reduction in the clinical setting, and infection control.
**B9**	Demonstrate critical thinking (evidence-informed decision-making skills, evidence-based practice skills) Examples include applying evidence-informed decision-making skills, evidence-based practice skills that include patient values, obtaining and evaluating new information, and applying appropriate new information to clinical practice setting.
**B10**	Demonstrate business management skills (use current technology, effectively run a practice, record keeping, patient confidentiality/privacy) Examples include using current technology, sustainable and ethical business skills, running a practice in a private, group, or integrated setting, accurate record keeping, and maintaining patient confidentiality/privacy.
**Code**	**Student Personal, Behavioral Competence**
	Personal/behavioral competence "the ability to adopt appropriate, observable behaviors in work-related situations."
**C1**	Demonstrate personal competence (self-care, self-improvement, acquisition of new knowledge and skills, technology and information literacy, life long learning skills) Examples include demonstrating self-care (mental, physical), self-improvement, acquiring new knowledge and skills, maintaining technology and information literacy, and lifelong learning skills.
**C2**	Demonstrate patient/person competence (person-centered care, empathy, cultural competence/diversity, professional/caring interpersonal communication and behaviors with patients) Examples include showing skills related to person-centred care, people-centred care, empathy, cultural competency, diversity/equity/ inclusion, professional/caring interpersonal communication and behaviours with patients, patient families and caregivers.
**C3**	Demonstrate professional competence (interpersonal communication and collaboration with other providers, team-based care) Examples include exhibiting interpersonal communication and collaboration with other providers, team-based care, communication and interactions with community and profession, functioning in interprofessional settings.
**Code**	**Student Values, Ethical Competence**
	Values/ethical competence "the possession of appropriate personal and professional values and the ability to make sound judgements based upon these in work-related situations."
**D1**	Demonstrate professionalism (honesty, integrity, ethics, leadership) Examples include demonstrating honesty, integrity, ethics, and leadership.
**D2**	Demonstrate practice competence (regional jurisprudence, legal, regulatory compliance, licensure, self-regulation of profession) Examples include demonstrating sound jurisprudence practices, legal and regulatory compliance, maintaining licensure, and self-regulation within the chiropractic profession.
**Code**	**Chiropractic Program**
	Information related to chiropractic programs, infrastructure, and delivery of chiropractic education
**E1**	Program Governance and Administration (demonstration of governance effectiveness, effective leadership, administration structure, demonstrate achieving mission of program) Examples include: The program demonstrates effective governance, effective leadership, and the inclusion of stakeholders. The program has an effective administration structure and processes to achieve the mission of the chiropractic program. The program acts with transparency, integrity, professionalism, equity, and inclusion.
**E2**	Program Assessment and Quality Improvement (improvement/quality/effectiveness of program; program evaluates its operations, improves performance through institutional and program effectiveness processes) Examples include: The program demonstrates processes for improvement/quality/effectiveness, evaluation of operations, improvement of performance through institutional and program effectiveness processes. The program demonstrates the use of current best practices for program assessment and quality improvement in higher education. Assessment of patient care in the clinical teaching/learning environment meets current healthcare best practices.
**E3**	Program Ethics (integrity on program level, adherence to ethical standards, policies/procedures, functions, governing body, administration) Examples include: The program demonstrates integrity on a program level. The program adheres to ethical standards, ethics policies/procedures, ethics governing body and administration; and shows processes for resolution of ethics issues using best practices.

**E4**	Program Methods (innovations, teaching methods, education paradigms, such as competency-based, interprofessional, integrative, problem-based learning, etc) Examples include: The program uses current best practices in education delivery, teaching and learning methods and provides best practices in education delivery paradigms for chiropractic education and training.
**E5**	Program Faculty/Staff (program development or support of faculty teaching methods, teaching, expertise, research, scholarship, service, improvement) Examples include: The program hires and supports qualified academic and clinical faculty, and administrative and technical staff. The program has resources for effective teaching methods, supports faculty development of expertise, demonstrates scholarship (publication, research, teaching), service, faculty/staff development, and the infrastructure to support faculty and staff.
**E6**	Program Student Support (program characteristics that support student experience, equity/inclusion, admissions, success, performance, learning) Examples include: The program administration and services support the student learning experience, diversity/equity/ inclusion, student admissions processes, support for academic success and performance, academic advisement/tutoring, person/learner-centered practices.
**E7**	Program Curriculum (curriculum development, assessment/monitoring of program content, curriculum, or competencies overall) Examples include: The program has a clearly articulated curriculum, which is up to date with current content and teaching/learning methods. The program has a process for assessment/monitoring of program content/competencies and curriculum development.
**E8**	Program Student Assessment (evaluation of performance data that includes, licensing exam success rates, program completion rates, students demonstrate meta-competencies) Examples include: The program evaluates student competencies/meta-competencies. The program assesses student and graduate performance data, which may consist of qualifying exam success rates and program completion rates.
**E9**	Program Resources (resources needed to support program success, financial stability/management, infrastructure, facilities, technology, expertise, human resources) Examples include: The program maintains a sustainable educational environment with financial, physical, human, and administrative resources needed to support program success. The program provides appropriate classroom, lab, technological, and clinical learning environments. The program shows financial stability/management, infrastructure, technology, facilities that support learning, human resources, and learning resources.
**E10**	Program Accreditation and Requirements (process of accreditation for chiropractic or regional accreditation, demonstration of how it is meeting program aims, goals, mission, meet regional legal requirements) Examples include: The program obtains and maintains relevant and required chiropractic and/or regional accreditation. The program demonstrates how the program is meeting accreditation requirements and jurisdictional regulatory requirements.

Cheetham G, Chivers GE. Professions, competence and informal learning: Edward Elgar Publishing; 2005. Quotations from pages 87 and 88.

Appendix C

Description of the modified Delphi consensus process

Modified- Delphi Round 1

For the first consensus round, the objective was to identify the percent agreement and gather initial corrections or modifications to the seed codes. The spreadsheets were sent to each panelist and included the following columns: text of each accreditation item, proposed code, area for the panelist to enter a suggested change or addition, and area for the panelist to enter an explanation and comment. Panelists were given two weeks to complete their reviews.

After the first-round responses were collected, the suggested modifications and comments were entered into a single Excel spreadsheet. Panelist comments and suggestions were collated and used by the principal investigator (CDJ) to edit the codes to create the sheet for the second round. Comments or corrections for any item were collated into one column using the Excel concatenate function and thus did not include any panelist personal identifiers. Comments were gathered and combined, and the percentage agreement for each item was calculated. Items that required no corrections or suggestions were given "100% agreement" status, and no further review was needed for those items. The data were used by the principal investigator (CDJ) to update the codes to be included in the form for the next consensus round.

Modified- Delphi Round 2

The round two spreadsheets contained the updated codes and the percentage agreement for each item. Feedback was provided to panelists through the updated coding spreadsheets and the percent agreement scores. The results were blinded, so no panelist knew who agreed or commented on any item. The only person who was aware of panelist identities was the principal investigator, who kept track of participation and recorded responses of the panelists.

The experts were asked to perform the same task as they did in round one, which was to either agree or, if they disagreed, to offer an amendment with comments explaining the suggested change. Items that had 100% agreement from the first round were still included to show context and retain meaning for surrounding codes. After the second-round responses were collected, the suggested modifications and comments were entered into a single spreadsheet in Excel. The findings were processed in the same manner as round one.

Modified- Delphi Round 3

The third-round spreadsheets included the updated codes and columns clarifying each code. Items that had 100% agreement from the prior rounds were not modified but were included to show context and retain meaning for surrounding codes. The experts were asked to perform the same task as they did with the prior two rounds, which was to agree or disagree and to offer their corrections with explanation for the requested change.

Nominal Group Technique Round 4

The result of the third round showed that 24 of 249 student competency standards and 38 of 136 program statements had less than 80% agreement or had opposing or confusing responses from the panelists. Therefore, we chose to complete a fourth round using nominal group technique. Those with leadership experience on accreditation boards (LAW, CL, CY) from 3 different regions were invited by the principal investigator (CDJ) to participate in a virtual meeting. The objective of the fourth round was to resolve disagreements on the remaining items. The items were reviewed and discussed during the meeting until a final decision on the coding was reached for each item.

Appendix D

Demographic characteristics of panelists and countries represented

Characteristics		
Sex		
	Male	13
	Female	8
Years employed in academia	(483 years total, average 23 years per expert)	
	0 to 10	5
	11 to 20	3
	21 to 30	8
	31 to 40	3
	41 +	2
Education	Doctor of chiropractic or other chiropractic degree	19
	Doctorate in education	3
	PhD or other doctorate	12
	Master's degree in education	6
	Master's degree (other, not chiropractic)	12
	Bachelor's degree (other, not chiropractic)	12
Amount of time assigned to professional roles	Teaching in lecture, lab, online (delivery of lecture/lab, course preparation, open lab teaching, office hours, course administration)	20.9%
	Clinical teaching (supervising learners with patient care, clinical administrative work, mentoring in clinical setting)	11.3%
	Research (completing research studies, writing manuscripts, scholarly activities, peer review, grant writing)	10.1%
	Administrative duties (supervising other academics, faculty, or staff; management of program functions, ethics, admissions)	50.1%
	Clinical practice - seeing patients not in an academic setting	7.5%

Country of author representation	Estimated number of chiropractors in that country (Stochkendahl, 2017)
United States of America and regions	77,000
Canada	8500
Australia	5277
United Kingdom of Great Britain and Northern Ireland	3,200
Brazil	1000
South Africa	792
New Zealand	600
Malaysia	160
Estimated total chiropractors globally	105,000

Appendix E

Percent agreements for coding for the four review rounds (three modified-Delphi and fourth round nominal group technique). All 21 panelists participated in the first three rounds. The fourth round was completed by four panelists.

**Student Competencies Agreement**	**Round 1**	**Round 2**	**Round 3**	**Round 4**
Average	91%	90%	93%	98%
Median	90%	95%	95%	100%
Mode	95%	100%	100%	100%
Range low to high score	57 to 100	33to 100	67 to 100	81 to 100
**Program Agreement**	**Round 1**	**Round 2**	**Round 3**	**Round 4**
Average	87%	83%	95%	99%
Median	90%	86%	95%	100%
Mode	95%	100%	100%	100%
Range low to high score	33 to 100	29 to 100	71 to 100	90 to 100

Appendix F

Statistical Formulations. The following statistical formulations correspond to the analyses performed:

$$Y_{ij} = \mu + \alpha_i + \beta_j + \epsilon_{ij}$$

where μ is the grand mean, α_i is the effect of level *i* of factor A (Program effect), β_j is the effect of level *j* of factor B (Code effect), and $\epsilon_{ij} \sim N(0, \sigma^2)$.

In the new model Y_{ij} is the observed response (adjusted percentage) for the ith level of factor A and j^{th} level of factor B.

Model 1: The assumptions for the two-way additive ANOVA were assessed using standard diagnostic tests. Residual normality was supported by the Shapiro–Wilk test (W = 0.985, p = 0.326), indicating no significant deviation from normality, which was further confirmed by visual inspection of the Q–Q plot, where residuals aligned closely with the reference line. Homogeneity of variances across the four categories (CCE, CCEA, ECCE, FCC) was confirmed using Levene's test (F = 0.221, p = 0.882), suggesting that the variance of the percentage scores was consistent across groups. These results support the validity of the ANOVA model's assumptions.

Normality of residuals was evaluated using the Shapiro–Wilk test, which yielded W = 0.985, p = 0.326, indicating no significant departure from normality. Additionally, visual inspection of the Q–Q plot of residuals confirmed this finding, with residual points closely aligning along the diagonal reference line. Homogeneity of variances across the four categories (CCE, CCEA, ECCE, CCEC) was assessed using Levene's test, which yielded F = 0.221, p = 0.882, indicating that the assumption of equal variances is met.

Model 2: The assumptions underlying the additive ANOVA model were assessed and found to be adequately met. The residuals were approximately normally distributed, as indicated by the Shapiro–Wilk test (W = 0.978, p = 0.604), and further supported by the Q–Q plot, which showed no substantial deviations from the normality line. Additionally, Levene's test for homogeneity of variances was not significant (F = 0.815, p = 0.494), indicating that the variances across the four categories (CCE, CCEA, ECCE, FCC) were sufficiently equal.

Normality of residuals was evaluated using the Shapiro–Wilk test, which yielded W = 0.978, p = 0.604, indicating no significant departure from normality. Additionally, visual inspection of the Q–Q plot of residuals confirmed this finding, with residual points closely aligning along the diagonal reference line. Homogeneity of variances across the four categories (CCE, CCEA, ECCE, CCEC) was assessed using Levene's test, which yielded F = 0.815, p = 0.494, indicating that the assumption of equal variances is met.

Q-Q Plot Model 1

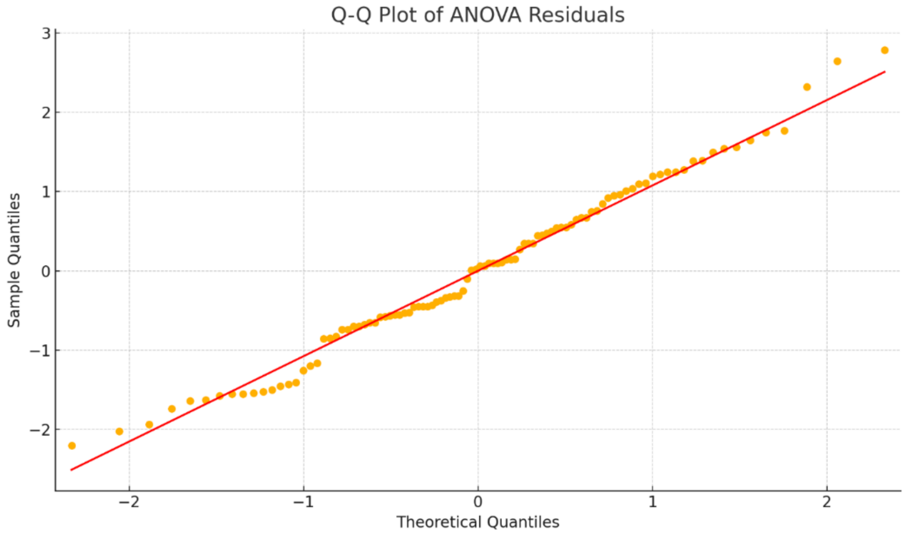

Q-Q Plot Model 2

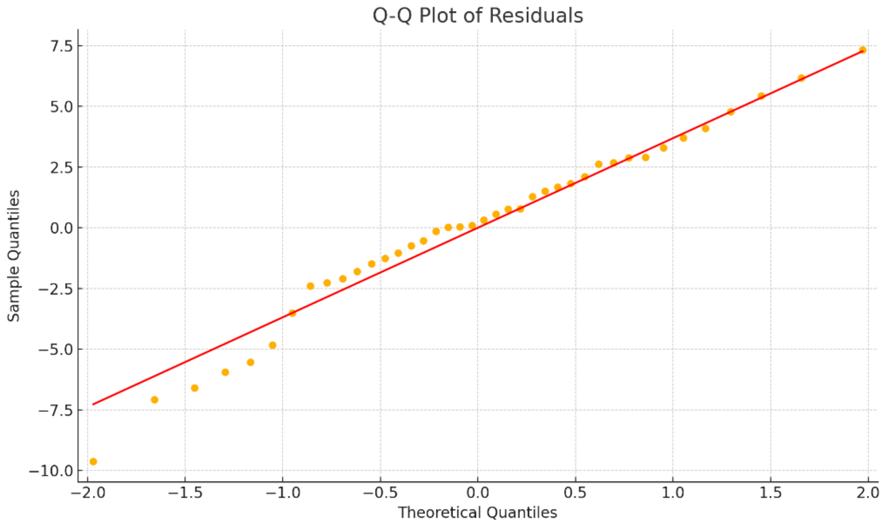

Appendix G

This table shows the proportion of codes represented within each individual accrediting agency for student competencies and program standards. The frequency is reported within the individual accreditation agency and is not combined with the others. This figure represents a global impression of the content emphasized in chiropractic programs worldwide.

Student Competency Code	Code Description	CCE	CCEA	ECCE	CCEC	Combined AVR
A6	Knowledge of chiropractic care (e.g., information related to safety and effects of chiropractic care)	8.5%	6.4%	9.7%	7.1%	8%
A10	Knowledge of other factors (e.g., clinical impacts, other health professions' treatments)	6.8%	8.3%	8.6%	5.8%	7%
A1	Knowledge of chiropractic (e.g., principles, theories, history, chiropractic in the healthcare system)	4.2%	4.5%	6.5%	8.6%	6%
A4	Knowledge of health (e.g., determinants of health)	8.5%	6.4%	4.3%	3.7%	6%
A7	Knowledge of patient/person needs (e.g., patient choices/needs)	5.7%	5.4%	5.4%	7.7%	6%
C2	Demonstrate patient/person competence (e.g., person-centered care, empathy)	4.8%	5.4%	5.4%	8.3%	6%
A5	Knowledge of evaluation (e.g., information needed for patient assessment)	6.5%	4.5%	5.4%	3.4%	5%
B2	Demonstrate clinical reasoning skills for the individual patient (e.g., formulate diagnosis/clinical impression)	6.2%	4.5%	4.3%	4.3%	5%
B3	Demonstrate health management (e.g., management plan, monitor patient response)	5.7%	6.1%	3.2%	3.4%	5%
B4	Perform communication (e.g., report of findings, obtain patient consent, discuss patient values)	3.7%	5.7%	3.2%	5.2%	5%
B9	Demonstrate critical thinking (e.g., evidence-informed decision-making skills)	5.4%	4.5%	2.2%	5.5%	5%
C3	Demonstrate professional competence (e.g., interpersonal communication and collaboration)	4.5%	6.1%	3.2%	5.5%	5%
A3	Knowledge of abnormal (e.g., structure and function, pathology)	4.0%	4.1%	5.4%	2.5%	4%
A2	Knowledge of normal (e.g., structure and function, anatomy, physiology, biokinematics)	4.0%	3.5%	3.2%	1.5%	3%
B1	Perform assessment of the individual patient (e.g., patient-relevant history, examination)	4.2%	2.5%	3.2%	2.8%	3%
B8	Demonstrate safety (e.g., patient safety, provider safety, hygiene, risk reduction)	3.4%	3.2%	2.2%	1.8%	3%
B10	Demonstrate business management skills (e.g., use current technology, effectively run a practice)	2.3%	2.9%	2.2%	3.1%	3%
D1	Demonstrate professionalism (e.g., honesty, integrity, ethics, leadership)	2.0%	3.2%	5.4%	4.0%	3%
D2	Demonstrate practice competence (e.g., regional jurisprudence, legal, regulatory compliance)	3.1%	2.5%	3.2%	3.4%	3%
A8	Knowledge of special populations (e.g., emergency care, pediatrics, geriatrics)	2.0%	3.2%	1.1%	1.5%	2%
A9	Knowledge of research (e.g., critical appraisal and application of research to health care)	0.8%	2.2%	4.3%	3.7%	2%
B7	Demonstrate therapeutic education, instructions (e.g., eg, self-care, home exercises, injury prevention)	1.1%	1.6%	2.2%	2.5%	2%
C1	Demonstrate personal competence (e.g., self-care, self-improvement, acquisition of new knowledge and skills)	0.8%	2.9%	3.2%	2.5%	2%
B5	Perform manual therapies (e.g., adjusting /manipulation/ mobilization, manual soft tissue therapies)	1.1%	0.3%	1.1%	1.5%	1%
B6	Perform supporting therapies (e.g., non-manipulative therapies)	0.6%	0.3%	2.2%	0.9%	1%
**Program Standard Code**	**Code Description**	**CCE**	**CCEA**	**ECCE**	**CCEC**	**Combined AVR**
E7	Program Curriculum (e.g., curriculum development, assessment/monitoring of program content)	6.3%	14.1%	23.2%	20.0%	15%
E1	Program Governance and Administration (e.g., governance effectiveness, effective leadership)	12.5%	7.8%	15.9%	14.3%	13%
E2	Program Assessment and Quality Improvement (e.g., improvement/quality/effectiveness of program)	13.8%	10.9%	13.0%	14.3%	13%
E9	Program Resources (e.g., resources needed to support program success)	17.5%	4.7%	11.6%	11.4%	12%
E5	Program Faculty/Staff (e.g., program development or support of faculty teaching methods)	13.8%	7.8%	10.1%	8.6%	11%
E8	Program Student Assessment (e.g., evaluation of performance data)	8.8%	14.1%	5.8%	8.6%	9%
E4	Program Methods (e.g., teaching methods, education paradigms)	2.5%	10.9%	10.1%	8.6%	8%
E6	Program Student Support (e.g., support student experience, equity/inclusion, admissions, success)	10.0%	12.5%	5.8%	0.0%	8%
E10	Program Accreditation and Requirements (e.g., process of accreditation for chiropractic or regional accreditation)	7.5%	9.4%	4.3%	5.7%	7%
E3	Program Ethics (e.g., integrity on program level, adherence to ethical standards, policies/procedures)	7.5%	7.8%	0.0%	8.6%	6%

CCE = Council on Chiropractic Education
ECCE = European Council on Chiropractic Education
CCEA = Council on Chiropractic Education Australasia
CCEC = Council on Chiropractic Education Canada
